# Glial Derived TGF-β Instructs Axon Midline Stopping

**DOI:** 10.3389/fnmol.2019.00232

**Published:** 2019-09-27

**Authors:** Neta Marmor-Kollet, Itai Gutman, Noa Issman-Zecharya, Oren Schuldiner

**Affiliations:** Department of Molecular Cell Biology, Weizmann Institute of Sciences, Rehovot, Israel

**Keywords:** *Drosophila*, axon, midline, glia, axon stopping, mushroom body, develepment, axon-glia interaction

## Abstract

A fundamental question that underlies the proper wiring and function of the nervous system is how axon extension stops during development. However, our mechanistic understanding of axon stopping is currently poor. The stereotypic development of the *Drosophila* mushroom body (MB) provides a unique system in which three types of anatomically distinct neurons (γ, α’/β’, and α/β) develop and interact to form a complex neuronal structure. All three neuronal types innervate the ipsi-lateral side and do not cross the midline. Here we find that Plum, an immunoglobulin (Ig) superfamily protein that we have previously shown to function as a TGF-β accessory receptor, is required within MB α/β neurons for their midline stopping. Overexpression of Plum within MB neurons is sufficient to induce retraction of α/β axons. As expected, rescue experiments revealed that Plum likely functions in α/β neurons and mediates midline stopping *via* the downstream effector RhoGEF2. Finally, we have identified glial-derived Myoglianin (Myo) as the major TGF-β ligand that instructs midline stopping of MB neurons. Taken together, our study strongly suggests that TGF-β signals originating from the midline facilitate midline stopping of α/β neuron in a Plum dependent manner.

## Introduction

During development of the nervous system, individual neurons send axons and dendrites to form intricate neuronal circuitry whose precise connectivity underlies the proper function of the brain. Roger Sperry, who pioneered the inception of the chemoaffinity hypothesis (Sperry, 1963), postulated that the proper development of neuronal connectivity is orchestrated by a complex system of molecular cues. This hypothesis has since been solidified as a wide variety of neuronal guidance molecules have been identified (Tessier-Lavigne and Goodman, 1996; Dickson, 2002; Kolodkin and Tessier-Lavigne, 2011). These include molecules that can act from a distance and those that act only in close contact. While most of the molecules, such as slits and netrins, were initially identified as either being attractive or repulsive, it is becoming clear today that most if not all molecules can have both attractive and repulsive roles, depending on the context and identity of the interacting receptors (Kolodkin and Tessier-Lavigne, 2011; Kaplan et al., 2014). However, while there is a growing understanding of the mechanisms that promote axon growth and guidance, much less is known about the mechanisms that induce axon stopping.

The *Drosophila* mushroom body (MB) is a bilaterally symmetrical central nervous system (CNS) neuropil structure that is mostly involved in associative learning and memory (reviewed in Heisenberg, 2003; Aso et al., 2014). It is comprised of three types of intrinsic neurons (γ, α’/β’, and α/β), sequentially born from four identical neuroblasts per hemisphere (Crittenden et al., 1998; Lee et al., 1999). Each neuron type exhibits a unique morphology, connectivity and function (Trannoy et al., 2011; Aso et al., 2014). Interestingly, all three MB neuronal types are confined to one hemisphere and do not cross the midline. Therefore, the stereotypic anatomical morphology of specific MB neurons and its relative simplicity offer a unique model to study axon guidance, branching and stopping.

Several studies have reported of mutations in which α/β neurons fail to stop at the midline and therefore cross to the contralateral hemisphere. This phenotype has been attributed to mutants in both intrinsic and extrinsic cues (Moreau-Fauvarque et al., 1998; Michel et al., 2004; Kobayashi et al., 2006; Grillenzoni et al., 2007; Kurusu and Zinn, 2008). Despite these findings, the cellular and molecular mechanisms responsible for axonal stopping remain mostly elusive. One of the best-documented examples in which α/β-axons fail to stop at the midline is in TGF-β type I receptor Baboon (babo) mutants. Ng (2008) has used the mosaic analysis with a repressible cell marker (MARCM) technique to show that MB neuroblast clones mutant for babo exhibit β-axon midline crossing. Additionally, he showed that in this context babo functions in a non-canonical fashion, signaling *via* LIMK rather than Smads. However, the identity of the TGF-β ligand, its source, and the precise cellular mechanisms are still not known.

In previous work we showed that Plum, an immunoglobulin super family (IgSF) trans-membrane protein, is required to promote pruning of MB γ-neurons, most likely functioning as a TGF-β accessory receptor (Yu et al., 2013). Here, we explore the role of Plum in the midline stopping of α/β neurons. Interestingly, we uncovered that glial-derived TGF-β ligands instruct midline stopping of MB α/β-axons.

## Materials and Methods

### *Drosophila* Strains

The *plum*^Δ1^ allele and the following Plum transgenes: UAS-Plum::Flag, UAS-Plum^Δcyt^::Flag, UAS-Plum^ΔFNIII(1-5)^::Flag, UAS-Plum^ΔIG1-4^::Flag, UAS-Plum^ΔIg1-3^::Flag, UAS-Plum^ΔIg4^::Flag, UAS-Plum^ECD^::Flag and UAS-Plum^ECD^::TM-CD8::Flag were previously described (Yu et al., 2013). G0050-Gal4 was kindly provided by A.S. Chiang. *UAS-Myo* RNAi (31200 and 31114), GMR71G10-Gal4, GMR44E04-Gal4 (originally made as part of the FlyLight project at the Janelia farm, Rubin lab), c305a-Gal4, c155-Gal4, Repo-Gal4 and *UAS-Plum* RNAi (TRiP.HMC05055, originally made by DRSC) were obtained from the Bloomington *Drosophila* Stock Center (BDSC).

### Construction of *plum*^V11X^ CRISPR Mutant Flies

Two guide RNA sequences were cloned into pCFD4 plasmid using Transfer-PCR as described previously (Port et al., 2014; Meltzer et al., 2019). The pCFD4^Plum^ plasmid was injected into attP86Fb landing site using ϕC31 integration (BestGene). Injected flies were crossed with nanos-Cas9 flies (Bloomington stock #54591). After two generations, single males were crossed with balancers and checked for deletions/indels using specific primers. gRNA plum specific sequences (5′-3′):

Forward: CAATCAATTGAATCACAAAG.

Reverse: TCACCCATTGGGATCCACCT.

### Genotypes Abbreviations

Full genotypes are detailed in the figure legends and are abbreviated as follows: hsFlp is *y, w, hsFlp122*; CD8 is *UAS-mCD8::GFP*; UAS-Plum^FL^ is *UAS-Plum::Flag*; UAS-Plum^Δcyt^ is *UAS-Plum*^Δcyt^::Flag; UAS-Plum^ΔFNIII(1-5)^ is *UAS-Plum*^ΔFNIII(1-5)^::Flag; UAS-Plum^ΔIG1-4^ is *UAS-Plum*^ΔIG1-4^::*Flag*; UAS-Plum^ΔIg1-3^ is *UAS-Plum*^ΔIg1-3^::*Flag*; UAS-Plum^ΔIg4^ is *UAS-Plum*^ΔIg4^::Flag; UAS-Plum^ECD^ is *UAS-Plum*^ECD^::Flag; UAS-Plum^ECD^::TM-CD8 is *UAS-Plum*^ECD^::TM-CD8::Flag; plum^Δ1^ is *82B, plum*^Δ1^; plum^V11X^ is *82B, plum*^V11X^; 40A, and 82B are FRTs on 2L and 3R, respectively; 201Y is *201Y-Gal4*; OK107 is *OK107-Gal4*; R71G10 is *GMR71G10-Gal4*; R44E04 is *GMR44E04-Gal4*; Repo is *Repo-Gal4*; c155 is *c155-Gal4*; TIFR is *TIFR-Gal4*; G0050 is *G0050-Gal4*; c305a is *c305a-Gal4*; Gal80 is *TubP-Gal80*; UAS-plum^RNAi^ is *TRiP.HMC05055* from DRSC/TRiP Functional Genomics Resources; UAS-Myo^RNAi^ (31200 or 31114) are either *TRiP.JF01717* or *TRiP.JF01587*, respectively, from DRSC/TRiP Functional Genomics Resources.

### MARCM and Antibody Conditions

MB MARCM neuroblast clones were generated by heat shocking newly hatched larvae and examined as described previously (Lee et al., 1999).

Antibody staining conditions were as follows: mouse monoclonal anti-FasII (ID4; 1:25); anti-Trio (9.4A; 1:100), both from the Hybridoma bank (DSHB); chicken polyclonal anti GFP (1:500; Aves); rat polyclonal anti mCD8 (1:200; Invotrogen MCD0800). FITC (ENCO), Alexa488 or Alexa647 (Invitrogen) conjugated secondary antibodies were used at 1:300. Brains were mounted in Slowfade (Invitrogen) and imaged on LSM710 or LSM800 confocal microscopes (Zeiss).

### Ranking and Statistical Analyses

**Figure 1:**
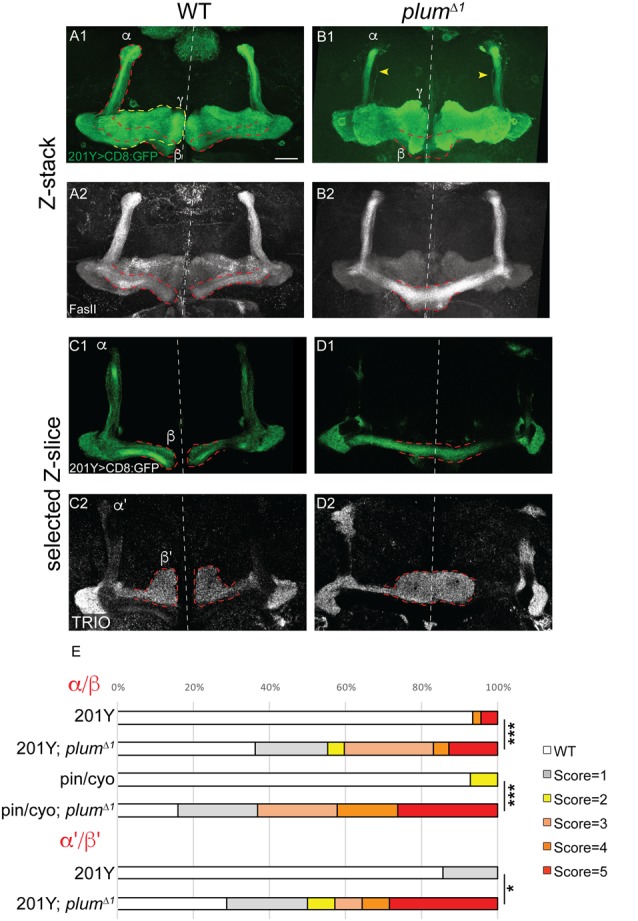
Mushroom body (MB) α/β and α’/β’ neurons over-extend beyond the midline in *plum*^Δ1^ homozygous brains. **(A–D)** Confocal Z-projections of WT **(A,C)** or *plum*^Δ1^ **(B,D)** brains expressing CD8::GFP driven by the 201Y-Gal4. mCD8::GFP (green, **A1–D1**); FasII (**A2,B2**, gray); TRIO (**C2,D2**, gray); Scale bars, 20 μm. **(A,C)** While in a WT brain the β-lobes **(A)** or β’-lobes **(C)** from both hemispheres terminate in a fair distance from each other and do not cross the midline, **(B,D)** the β- **(B)** or β’-axons **(D)** often cross the midline and sometime seem to fuse in *plum*^Δ1^ brains. Dashed lines demarcate the extent of the β or β’-lobes, yellow arrows in **(B)** indicate unpruned γ neurons. **(E)** Quantification of midline crossing phenotypes in (**A–D**; 5 being the most severe), as well as additional genotypes as mentioned below (for more information, see Supplementary Figure S1 and the “Materials and Methods” section). Genotypes: **(A)** 201Y, CD8/+ (*n* = 45). **(B)** 201Y, CD8/+; 82B, plum^Δ1^ (*n* = 42) *p*-value: *p* < 0.001. **(C)** 201Y, CD8/+; 82B, plum^Δ1^ (*n* = 14). **(D)** 201Y, CD8/+ (*n* = 7) *p*-value: *p* = 0.012. (Additionally, quantified in **E**): Pin/CyO (*n* = 14) and Pin/CyO; 82B, Plum^Δ1^ (*n* = 19), *p*-value: *p* < 0.001.

**Figure 2:**
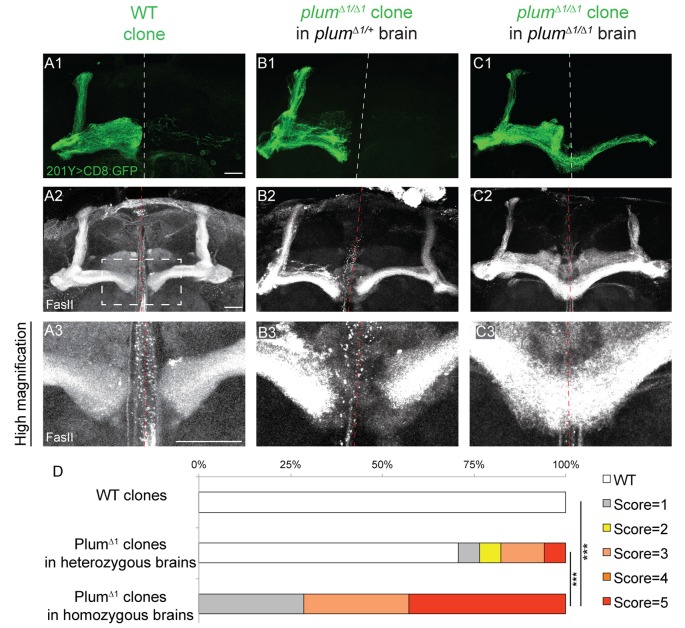
Plum is both autonomously and non-autonomously required for β-lobe midline stopping. **(A–C)** Confocal Z-projections of **(A)** WT mosaic analysis with a repressible cell marker (MARCM) neuroblast clone, **(B)** *plum*^Δ1^ MARCM clone in a *plum*^Δ1/+^ heterozygous brain or **(C)** *plum*^Δ1^ MARCM clone in a *plum*^Δ1^ homozygous mutant brains, labeled with 201Y-Gal4. CD8::GFP (green, **A1–C1**); FasII (gray, **A2–C3**). Scale bars, 20 μm. **(D)** Quantification of midline crossing phenotypes in **(A–C)**. *p*-values compared to *plum*^Δ1^ clone in *plum*^Δ1^ brain—WT clones: ***p < 0.001. *plum*^Δ1^ clone in heterozygous brain: *p* = 0.0074. Genotypes: **(A)** hsFlp, CD8/Y or +; 201Y, CD8/+; 82B, Gal80/82B (*n* = 10). **(B)** hsFlp, CD8/Y or +; 201Y, CD8/+; 82B, Gal80/82B, *plum*^Δ1^ (*n* = 17). **(C)** hsFlp, CD8/Y or +; Gal80, 40A, 201Y, CD8/40A; 82B, plum^Δ1^ (*n* = 7).

**Figure 3:**
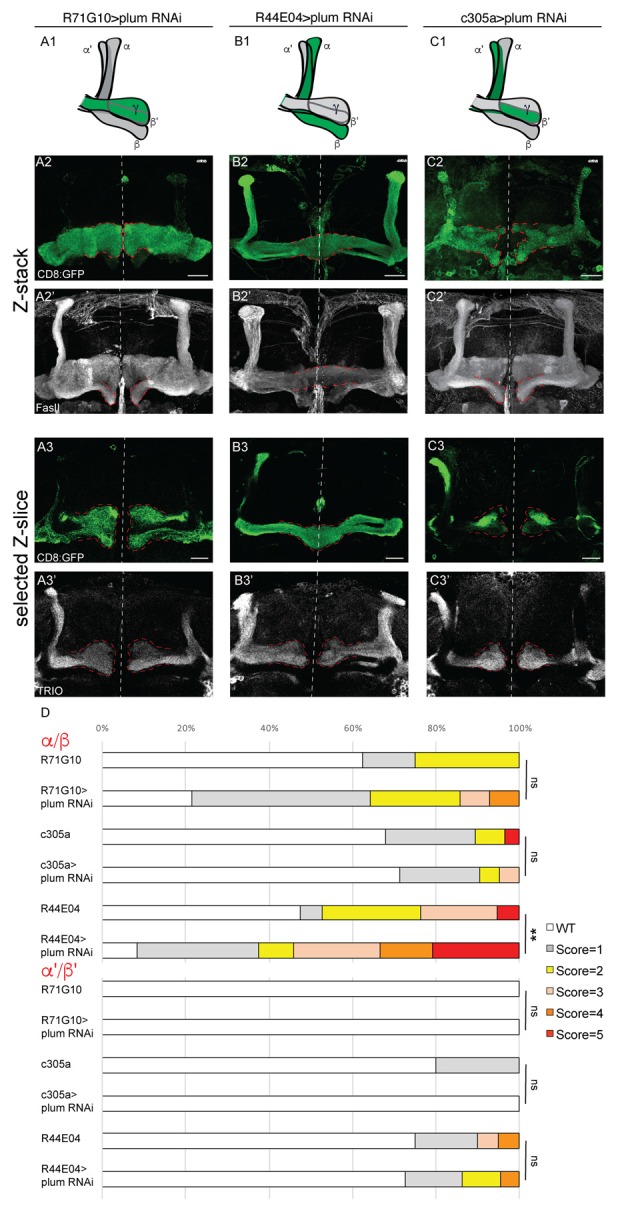
Plum is required in MB α/β neurons to initiate β-lobe midline stopping. **(A–C)** Confocal Z-projections of brains expressing Plum^RNAi^ specifically in γ neurons using the R71G10 driver **(A)**, in α/β neurons using the R44E04 driver **(B)** or in α’/β’ neurons using the c305a driver **(C)**. Top panel in each column represents a scheme of MB-axons, where the expression of each specific driver is illustrated in green. **(D)** Quantifications of the midline crossing phenotypes in (**A–C** and Supplementary Figure S4). *p*-values compared to controls in Supplementary Figure S4: in the case of R44E04 driven RNAi: **p = 0.003; ns=not significant. CD8::GFP (green, **A2–C3**); FasII (**A2’–C2’**, gray); TRIO (**A3’–C3’**, gray); Scale bars, 20 μm. Genotypes: **(A)** CD8/UAS-plum^RNAi^; R71/+ (**A2**: *n* = 14, **A3**: *n* = 18). **(B)** CD8/UAS-plum^RNAi^; R44/+ (**B2**: *n* = 24, **B3**: *n* = 22). **(C)** c305/UAS-plum^RNAi^; CD8/+ (**C2**: *n* = 21, **C3**: *n* = 8). Additionally, quantified in **(D)**: CD8; R71G10 (*n* = 16, 16, respectively). CD8; R44E04 (*n* = 38, 20, respectively). c305a; CD8 (*n* = 28, 20, respectively).

A single scorer performed ranking, in a double-blind manner. The ranking was performed by reviewing the entire z-stack. Ranking range was set as follows: WT = 0, β-lobes almost touching each other but not crossing the midline = 1 and β-lobes crossing the midline with increasing severity = 2–5. In Figures 1–3, five statistical significance was calculated by performing non-parametric Kruskal Wallis analysis of variance (ANOVA) test followed by, if necessary, *post hoc* pairwise Wilcoxon test. In Figure 6, statistical significance was calculated by performing one-tailed Wilcox test, and the *p*-values were adjusted by FDR correction.

**Figure 4:**
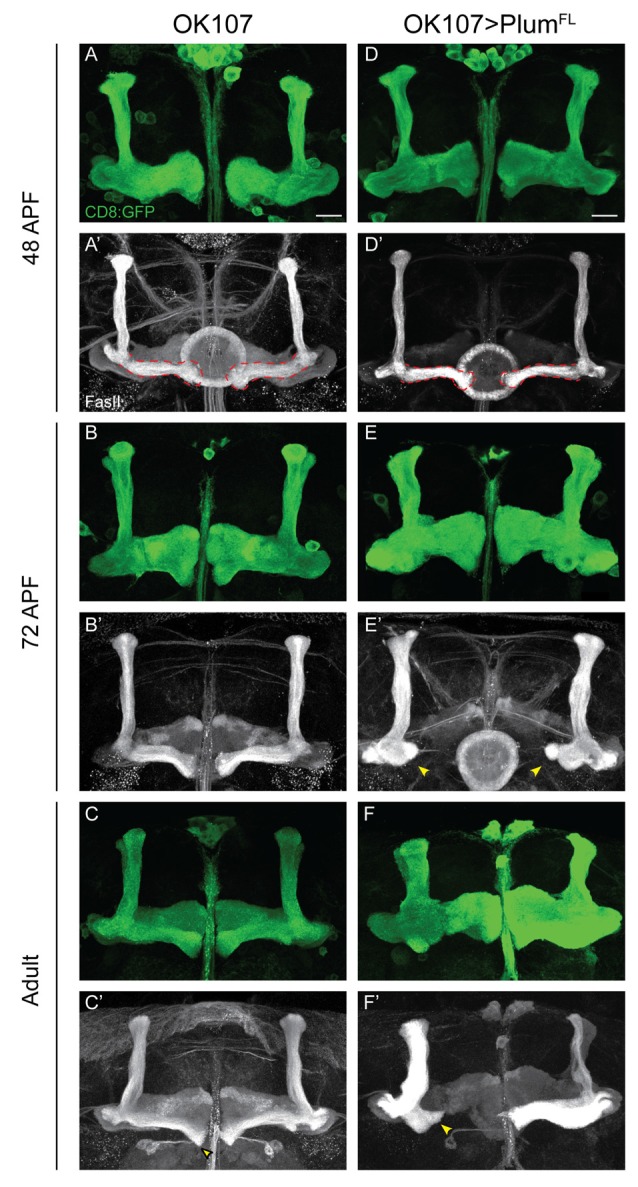
Plum over-expression within MB neurons is sufficient to induce β-lobe retraction. **(A–F)** Confocal Z-projections of **(A–C)** WT brains expressing CD8::GFP and **(D–F)** WT brains expressing CD8::GFP as well as Plum^FL^ driven by OK107-Gal4. Arrowheads in **(E,F)** depict aberrant growth of β-lobes. CD8::GFP (green, **A–F**); FasII (gray, **A’–F’**). Scale bars, 20 μm. Genotypes: **(A–C)** CD8/+; OK107 (*n* = 5, 5, 18, respectively). **(D–F)** UAS-Plum^FL^/CD8;; OK107 (*n* = 7, 15, 22, respectively).

**Figure 5:**
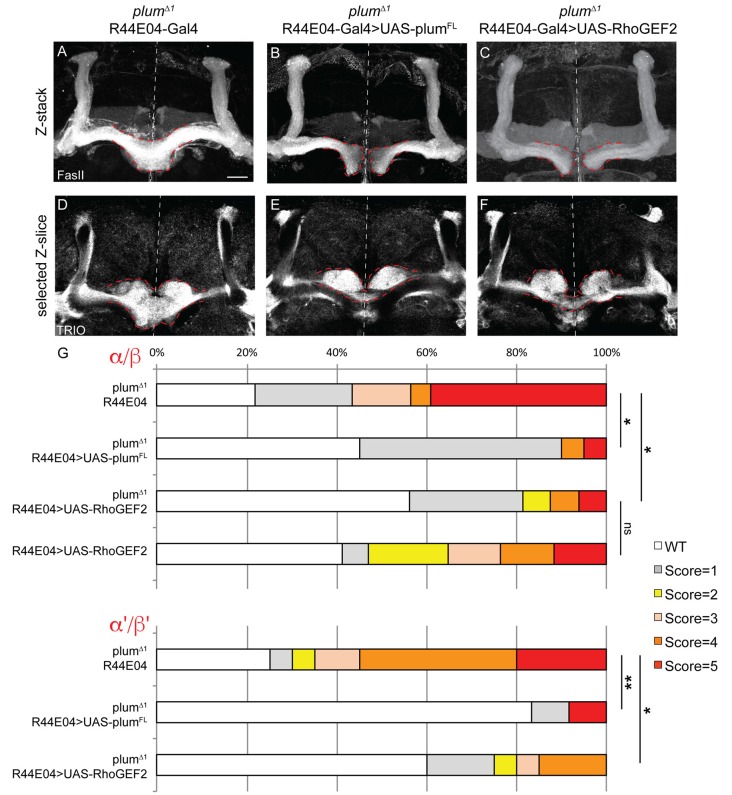
Plum acts through RhoGEF2 in α/β neurons to facilitate β-axon midline stopping. **(A–F)** Confocal Z-projections or selected Z-slices of adult **(A,D)** *plum*^Δ1^ brains expressing CD8::GFP driven by the R44-Gal4 driver, or brains additionally expressing **(B,E)** UAS-Plum^FL^ or **(C,F)** UAS-RhoGEF2. **(G)** Quantification of β-lobe midline crossing phenotype in **(A–F)**. *p*-values compared to plum^Δ1^ R44E04: plum^Δ1^ R44E04>Plum^FL^, *p = 0.038, **p=0.0098, for α/β and α’/β’, respectively. plum^Δ1^ R44E04>RhoGEF2, *p = 0.049, *p = 0.0123 for α/β and α’/β’, respectively. Dashed red lines demarcate the extent of the β-lobes. FasII (in **A–C**, gray); TRIO (in **D–F**, gray). Scale bars, 20 μm. Genotypes: **(A,D)** CD8::GFP/+; R44-Gal4, Plum^Δ1^/Plum^Δ1^ (*n* = 23, 39, respectively), **(B,E)** UAS-Plum^FL^/+; R44-Gal4, Plum^Δ1^/Plum^Δ1^(*n* = 20, 27, respectively), **(C,F)** UAS-RhoGEF2/+; R44-Gal4, Plum^Δ1^/Plum^Δ1^ (*n* = 16, 16, respectively).

**Figure 6:**
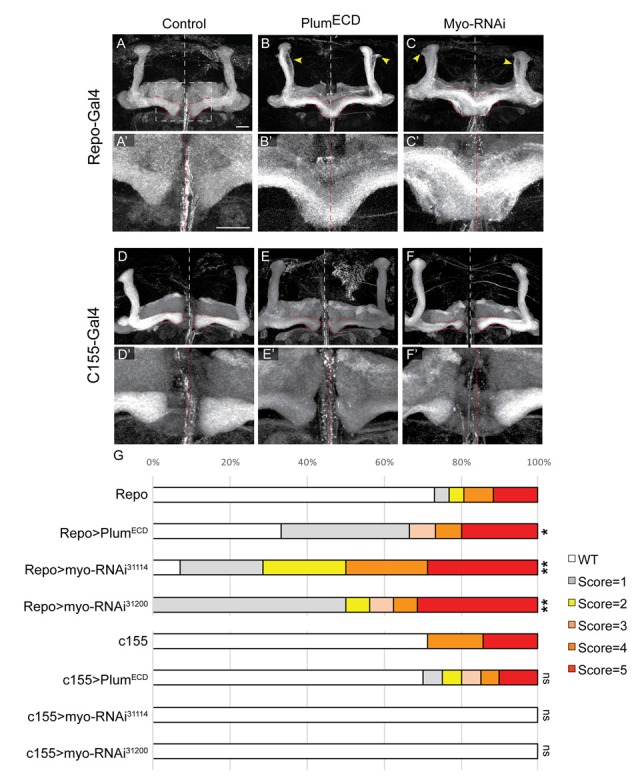
Glial-derived TGF-β ligand Myo is required for β-lobe midline stopping. **(A–F)** Confocal Z-projections of adult brains expressing **(A)** Repo-Gal4 as control or those expressing **(B)** Plum^ECD^ driven by Repo-Gal4 or **(C)** Myo^RNAi-31114^ driven by Repo-Gal4 or **(D)** those expressing c155-Gal4 as control or **(E)** Plum^ECD^ driven by c155-Gal4 or **(C)** Myo^RNAi-31114^ driven by c155-Gal4. Yellow arrows in **(B,C)** indicate unpruned γ neurons. Panels **(A’–F’)** are high magnification images of (**A–F**) FasII (gray). Scale bars, 20 μm. **(G)** Quantification of the β-lobes midline crossing in experiments. *p*-values compared to Repo: Repo-Gal4>Plum^ECD^, *p = 0.037; Repo-Gal4>Myo RNAi^31114^, **p = 0.0018; Myo RNAi^31200^, **p = 0.0012; *p*-values compared to c155: ns = not significant. By lanes from top to bottom in **(E)**: (1) Repo/+ (*n* = 26); (2) UAS-Plum^ECD^/Repo (*n* = 15); (3) Repo/UAS-Myo^RNAi-31114^ (*n* = 11); (4) Repo/UAS-Myo^RNAi-31200^ (*n* = 18); (5) c155 (*n* = 7); (6) c155(y or +); UAS-Plum^ECD^/+ (*n* = 20); (7) c155/(y or +); UAS-Myo^RNAi-31114^/+ (*n* = 10) and (8) C155/y or +; UAS-Myo^RNAi-31200^/+ (*n* = 9).

## Results

### Plum Is Required for Normal MB β-Lobe Development

We have previously focused on the role of *plum* during the development of MB γ neurons and have shown that it is cell-autonomously required for axon pruning (Yu et al., 2013). While analyzing *plum*^Δ1^ homozygous mutant brains, we noticed that another type of MB neurons, the late-born α/β neurons, were also defective. Specifically, when *plum* was homozygous mutant, we found that β-axons over-extended beyond the midline in 47% of the brains and often the β-lobes from both hemispheres seemed to fuse (17%; Figures 1A,B; see Supplementary Figure S1A and “Materials and Methods” section for ranking and statistics). This morphology was in contrast to WT brains where midline crossing was observed in only 7% of the brains while in the vast majority of the brains the β-lobe normally arrested at a considerable distance from the midline (compare Figure 1B with 1A, quantified in 1E). This low incidence of β-lobe midline crossing in WT brains is normal and consistent with previously described occasional wild-type β-lobe axon midline crossing (Moreau-Fauvarque et al., 1998; Michel et al., 2004).

The β-lobe midline crossing phenotype was even more pronounced when we looked at antibody staining for the FasII adhesion molecule (compare Figure 1B1 with 1B2). The difference in phenotype expressivity when looking at GFP vs. FasII expression might stem from the fact that FasII is highly expressed endogenously in most if not all β-axons while the expression of GFP driven by the 201Y-Gal4 driver is weaker and is limited to core β-lobe axons (Crittenden et al., 1998; Kurusu et al., 2002). Since *plum*^Δ1^ is a tailored deficiency that encompasses both *plum* as well as *CG5455* (Yu et al., 2013), we wanted to validate that the β-lobe midline crossing phenotype we observed is indeed caused by *plum* loss-of-function. Therefore, we generated a CRISPR/Cas9 mediated indel, *plum*^V11X^, a deletion of 10 bp resulting in a premature stop codon after 10 amino acids (Supplementary Figure S1B). A MARCM analysis of *plum*^V11X^ revealed that clones of MB γ neurons demonstrate a severe pruning defect (Supplementary Figures S1C,D), similar to *plum*^Δ1^ mutant clones (Yu et al., 2013). Indeed, in *plum*^V11X^ homozygous mutant brains, β-axons cross the midline in similar fashion as in *plum*^Δ1^ (Supplementary Figures S1E,F, quantified in Supplementary Figure S1G).

Plum is therefore required for midline stopping of α/β but not γ MB neurons. We next questioned whether Plum is required for the midline stopping of α’/β’ neurons. To test that, we stained *plum*^Δ1^ homozygous brains with TRIO antibody, which labels both γ and α’/β’ neurons, and were surprised to find that α’/β’ neurons also cross the midline in *plum*^Δ1^ brains (Figures 1C,D, quantified in 1E). These results suggest that in addition to its role in γ neuron pruning, Plum is required for normal midline stopping of β and β’-axons.

### Plum Is Required Within MB Neurons for β-Lobe Midline Stopping

We next questioned if Plum functions cell-autonomously, similar to its role during pruning. We, therefore, compared the morphology of *plum*^Δ1^ MB MARCM clones within *plum*^Δ1^ heterozygous or homozygous mutant brains (Figures 2B,C, respectively). Seventy percentage of β-axons in *plum*^Δ1^ mutant clones crossed the midline in homozygous brains, while only 20% crossed the midline in heterozygous brains and 0% control β-axons crossed the midline (Figures 2A,D for quantification).

The genotype of the mutant α/β MARCM clone in these experiments was identical with the only difference being the genotype of the surrounding cells. One possible interpretation of the difference between the phenotypes of *plum* mutant clone in heterozygous vs. homozygous brains (20% compared with 70%, respectively), could potentially be explained by protein perdurance. The clonal cells generated within *plum*^Δ1^ heterozygous brain, did express Plum before the FRT-mediated recombination occurred and therefore some *plum* mRNA and protein might still be present in low quantities. We think this interpretation is less likely due to the many cell divisions occurring in a neuroblast clone, resulting in constant dilution of Plum’s RNA and protein. Therefore, the most plausible interpretation suggests a non-autonomous role for Plum in the non-clonal cells, either in other α/β MB neurons, or in other MB or non-MB cells. Taken together, although Plum acts cell-autonomously to instruct pruning of MB-γ neurons (Yu et al., 2013), our results suggest that Plum midline stopping function also reflects a non-cell autonomous activity.

To investigate if the non-cell autonomous function of Plum is required in MB neurons, we knocked down *plum* expression specifically within MB neurons by expressing *plum* RNAi using a strong, pan-MB driver, OK107-Gal4 (Supplementary Figures S2A–D). Indeed, knocking down *plum* in all MB neurons resulted in 60% of the brains exhibiting severe β-lobe midline crossing (Supplementary Figures S2B,B’ compared to S2A,A’, quantified in S2E), and 45% of the brains exhibiting severe β-lobe midline crossing (Supplementary Figures S2D,D’ compared to S2C,C’, quantified in S2E), indicating that Plum expression is required within the MB for proper α/β or α’/β’ neuronal development.

### Plum Expression in α/β Neurons Is Required for β Lobe Midline Stopping

To specifically determine in which type of MB neurons Plum is required to induce midline stopping, we specifically knocked down *plum* in γ, α/β or α’/β’ neurons (Figure 3, see developmental expression pattern of α’/β’ and α/β-specific drivers in Supplementary Figures S3A–F). Knocking down *plum* in γ neurons (using R71G10-Gal4) did not affect midline stopping of either γ, β or β’-axons (Figure 3A quantified in 3D, compare to control in Supplementary Figure S4A). In contrast, knocking down *plum* only in α/β neurons (using the R44E04-Gal4) resulted in severe β lobe midline crossing in 30% of the brains (Figure 3B, quantified in 3D, compare to control in Supplementary Figure S4B), while the β’ lobes were unaffected and stopped at the midline (Figure 3B3’). Although the R44E04-Gal4 driver is not thought to be expressed in γ neurons, we also observed what seems to be a pruning defect when plum was knocked down using this driver (Figures 3B2’,B3’), perhaps due to a very low expression level of R44E04 also in γ neurons. Because α’/β’ neurons also cross the midline in *plum*^Δ1^ animals (Figure 1D), we expected Plum to have a role in their midline stopping as well. However, to our surprise, knocking down *plum* specifically in α’/β’ neurons (using both c305a-Gal4; Figure 3C, Supplementary Figure S4C; and G0050-Gal4; data not shown) did not induce midline crossing of β or β’-axons (Figures 3C2’,3C3’, respectively, quantified in 3D), however we cannot rule out the possibility that the α’/β’ drivers are either too weak or expressed only in a subset of α’/β’ neurons. In summary, our results indicate that Plum is important in α/β for their own midline stopping and also contribute to α’/β’ crossing to some degree.

### Plum Over-Expression in MB Neurons Is Sufficient to Induce β-Lobe Retraction

Our results show that Plum is required within MB neurons to induce midline stopping of β-axons. We, therefore, hypothesized that Plum over-expression within MB neurons could induce a premature stop signal and result in short α/β-lobes. Indeed, we found that over-expressing Plum by the strong, pan-MB driver OK107-Gal4, resulted in shorter β but not α or γ lobes (Figure 4F compare to 4C). We were intrigued to find, however, that β’ lobes length was not affected by over-expression of Plum, even in brains in which the β lobe has been retracted (Supplementary Figure S5). Because two branches of the same neuron form the α and β lobes, this result implies that the length of α and β branches of the α/β neurons is determined independently.

The manifestation of a short β-lobe in adult brains could be a result of at least two different scenarios. Either the growth of the β-lobe is impaired, leading to premature axon stopping as suggested by other studies focusing on β-lobe length (Ng, 2008; Abe et al., 2014), or the β-lobe could initially form normally but later undergo shortening either by axon retraction or fragmentation. To differentiate between these possibilities, we performed a developmental time course study of pupal brains over-expressing Plum driven by OK107-Gal4. To our surprise, we found that at 48 h after pupae formation (APF) the β-lobe seemed to develop normally and axons extend up to the midline (Figure 4D compare with 4A). However, as development proceeded, the β-lobe appeared progressively shorter at 60 h APF (data not shown) and even more so at 72 h APF (Figure 4E compare with 4B). As we did not observe axon fragments at any of the developmental time points, we conclude that β-lobe shortening by ectopic expression of Plum likely results by β-axon retraction.

In our previous work, we performed a detailed structure-function analysis and found that Plum functions in the context of MB γ neuron pruning as an accessory receptor, which can bind a ligand but cannot transduce a signal by itself. This observation was based on our findings that while Plum’s extracellular domains were required for its function in axon pruning, its cytoplasmic domain was not. To test if Plum functions similarly in α/β neurons, we performed a structure-function analysis. As expected, we found that deletions of specific extracellular domains that are required for pruning also abrogated Plum’s ability to induce β lobe retraction (Table 1). To our surprise, over-expression of a Plum transgene encoding for its extracellular domain fused to a CD8 transmembrane domain did not induce retraction (UAS::plum^ECD^::TM-CD8; *n* = 15; Table 1). Because over-expression of Plum lacking its cytoplasmic domain did induce axon retraction in 40% of the brains, these results suggest that in contrast to pruning, Plum transmembranal domain is required to induce retraction, likely *via* interactions with another signaling transmembrane receptor.

**Table 1:** Plum structure-function analysis.

Genotype	β retraction	WT
OK107>Plum^FL^ (*n* = 51)	75%	25%
OK107>Plum^ΔIg1-3^ (*n* = 36)	0%	100%
OK107>Plum^ΔIg1-4^ (*n* = 50)	0%	100%
OK107>Plum^ΔIg4^ (*n* = 26)	8%	92%
OK107>Plum^ΔFNIII(1-5)^ (*n* = 16)	0%	100%
OK107>Plum^Δcyt^ (*n* = 64)	41%	59%
OK107>Plum^ECD^::TM-CD8 (*n* = 15)	0%	100%

*Different Plum transgenes: either the full-length protein (Plum^FL^), or those lacking specific domains, as indicated, were driven specifically in MB neurons using the OK107-Gal4 driver. The percentage of brains that present β-lobe retraction or WT phenotypes is described*.

### Plum Acts Through the Downstream Effector RhoGEF2 in α/β Neurons to Facilitate β-Axon Midline Stopping

To further unravel Plum’s mode of action, we asked whether expression of Plum in MB α/β neurons within homozygous mutant brains is sufficient to rescue β-lobe midline crossing. We expressed different Plum rescue transgenes in MB α/β neurons using the R44E04-Gal4 driver, which is expressed in α/β neurons but also in few non-MB cells (Supplementary Figures S3D–F). indeed, expressing Plum in α/β neurons was sufficient to significantly rescue β-lobe over-extension phenotype (Figures 5A,B, quantified in 5G). Interestingly, expressing plum^Δcyt^, plum^ΔIG1-4^, plum^ΔIG-1-3^ or plum^ECD-TM:CD8^ could not rescue β-lobe crossing (Supplementary Figure S6), suggesting that unlike Plum’s domain requirement in γ axon pruning, where only the IG4 domain is important, in the context of β midline stopping, all of the tested extracellular, membranal and intracellular Plum domains are required. Interestingly, Babo, the TGF-β type I receptor, was shown to regulate axon midline stopping through a non-canonical Smad independent pathway, signaling *via* RhoGEF2 and LIMK (Ng, 2008). We, therefore, hypothesized that Plum might also act through this pathway in order to facilitate β-axon midline stopping. To test that, we expressed RhoGEF2 transgene in MB α/β neurons, in *plum*^Δ1^ homozygous brains. As expected, expressing RhoGEF2 specifically in α/β neurons was sufficient to significantly suppress the β-axon midline crossing phenotype (Figures 5A,C, Supplementary Figure S7, quantified in 5G). To our surprise, expressing either Plum^FL^ or RhoGEF2 specifically in α/β neurons was also sufficient to suppress the crossing of the β’-axons (Figures 5D–F, quantified in 5G), suggesting that Plum can mediate a non-cell autonomous interaction between these two neuronal types to induce midline stopping.

### The TGF-β Ligand Myoglianin Is Required for β-Axon Midline Stopping

Like *plum*, the TGF-β receptors—*babo, wit* and* punt*, were also previously shown to be required for normal β-lobe development (Ng, 2008). Loss of either one of these receptors results in β-lobe over-extension, while over-expression of a constitutively active version of Babo (Babo^CA^) results in short α and β-lobes. Furthermore, a recent study associated Myo as the activating ligand of Babo during pruning (Awasaki et al., 2011) and our own work has demonstrated genetic interaction between *plum* and *baboon* and between *plum* and *myo* (Yu et al., 2013). We were, therefore, interested to investigate whether TGF-β ligands, and perhaps Myo specifically, were involved in inducing a β-lobe stop signal.

We have previously shown that expressing a secreted form of Plum extra cellular domain (Plum^ECD^) has a dominant negative effect on MB γ neuron pruning, presumably by sequestering its ligand, which in the context of pruning is Myo. To test if secreted ligands are also involved in β-lobe stop signal, we first over-expressed a Plum^ECD^ in glia by using the pan-glial driver, Repo-Gal4. Indeed, glial over-expression of Plum^ECD^ resulted in β-lobe midline crossing in 34% of the brains (Figure 6B compare with 6A, quantified in 6G), while neuronal expression of Plum^ECD^ had no effect on midline crossing (Figure 6E compared with 6D, quantified in 6G), suggesting that Plum might interact with a secreted ligand.

The *Drosophila* genome encodes for three TGF-β ligands: Myoglianin (Myo), Dawdle (Daw) and Activin (dAct). Due to its known interaction with Plum in the context of axon pruning, we decided to focus on Myo; we over-expressed two different Myo-RNAi constructs in glial cells using the pan-glial driver Repo-Gal4 and in neurons using the pan-neuronal driver c155-Gal4. While 40% of β-lobes crossed the midline when we knocked down Myo expression in glia (Figure 6C compared with 6A, quantified in 6G), 0% crossed the midline when Myo was knocked down in neurons (Figure 6F compared to 6D, quantified in 6G) indicating that glial-derived Myo is required for proper β-lobe formation. The fact that only 40% of the β-lobes cross the midline in brains expressing Myo RNAi could suggest one of three options: Myo RNAi knockdown could be incomplete; Myo could be expressed in and secreted by cells other than glia; or Myo might function cooperatively with one of the other two TGF- β ligands, in determining β-lobes length. These results together demonstrate that Myo and possibly also Daw are the ligands required for β-axon stop signal at the midline.

## Discussion

In this study, we have uncovered a novel mechanism by which axon stopping is regulated. Using loss of function analyses combined with rescue and gain of function experiments, we found that Plum, an Ig superfamily plasma membrane receptor, is required for the midline stopping of α/β MB neurons, and acts through RhoGEF2. Additionally, we found that glial-derived TGF-β ligand Myo induces the midline stopping of α/β MB neurons.

Genetic evidence suggests that Plum is both required and sufficient in MB neurons to regulate midline stopping of β-axons (Figures 3, 4, respectively). However, the fact that *plum*^Δ1^ MARCM MB clones show a more severe β-lobe midline crossing in homozygous brains compared to heterozygous brains (Figure 2) suggests that Plum may also play an important non-cell autonomous role in β-lobe development. This is intriguing, especially in light of the fact that Plum is a cell surface receptor. Because in mosaic brains containing neuroblast MARCM clones, $\frac{3}{4}$ of the neurons are heterozygous while only $\frac{1}{4}$ of the neurons are homozygous to the mutation, one simple explanation would be that Plum functions cell-autonomously in α/β neurons but axon stopping might be a communal decision.

In addition to Plum being important in α/β neurons to instruct their midline stopping, we also observed midline crossing of α’/β’ neurons in *plum*^Δ1^ animals. While α’/β’ midline crossing has never been reported, many studies have identified conditions in which α/β neurons extend beyond the midline; yet the underlying cellular mechanisms remain unclear (Moreau-Fauvarque et al., 1998; Michel et al., 2004; Kobayashi et al., 2006; Grillenzoni et al., 2007; Kurusu and Zinn, 2008; Ng, 2008). To our surprise, although α’/β’ neurons cross the midline in *plum*^Δ1^ animals, when we attempted to knock down *plum* specifically in α’/β’ neurons (using two different drivers), there was no midline crossing in either α’/β’ or α/β neurons. This inconsistency may be due to technical reasons such as weak or late expression of the Gal4 driver or non-cell autonomous effects. Since expressing Plum or its downstream effector RhoGEF2 specifically in α/β neurons partially suppressed the midline crossing of both β and β’-lobes to a similar degree, this indeed hints for a non-cell autonomous role of Plum in the context of midline stopping or retraction. Since α’/β’ neurons are born during the late-larval stage, and already send their axons by the time the α/β neurons are born, this non-cell autonomous interaction needs to be further and carefully studied and evaluated. Therefore, we cannot rule out an additional role for Plum in α’/β’ neurons in β’ or β-axon midline stopping.

One potential strategy to restrict axon extension into the opposite hemisphere would be for the existence of a signal at the midline. Indeed, during embryonic development, the midline provides major instructive signals. These signals, such as slit, are mainly secreted by midline glia in the embryo (Sonnenfeld and Jacobs, 1994). How the developing adult brain continues to exhibit bilateral symmetry is much less studied. The transient interhemispheric fibrous ring (TIFR), a transient glia structure, was proposed as a source of signaling molecules required during pupal development. TIFR glial cells are required for olfactory receptor neurons (ORN) axons to project across the midline and establish the contralateral wiring in the antennal lobes (AL; Chen and Hing, 2008) and more importantly, were proposed to restrict β-lobes from crossing the midline, *via* an unknown molecular mechanism (Simon et al., 1998). We found that glial-derived Myo was required for normal stopping of α/β neurons. Therefore, our data suggest that Myo is at least one of these midline signals that instruct β-axon stopping. Unfortunately, the available tools prevented us from making a clear conclusion regarding the role of the TIFR-glia at this time but further studies are definitely warranted.

Our findings suggest that the Plum domains that are required for axon stopping (all tested extracellular, transmembranal and intracellular domains) are different from than those required for pruning (mainly the IG4). Nonetheless, our data suggest that Plum functions downstream to the TGF-β ligand myo and upstream to RhoGEF2, which was previously shown to mediate TGF-β type I receptor Babo (Ng, 2008). Therefore, our data is most consistent with Plum functioning as a TGF-β accessory receptor that facilitates TGF-β signaling. Whether the transmembranal domain of Plum is important to mediate its potential interaction with Babo or another unknown component of the receptor complex, and also the mechanistic differences in Plum function in axon pruning or stopping, remain to be further studied. Therefore, variations on TGF-β signaling between glia and neurons play diverse roles in neuronal development.

## Significance Statement

How axons stop growing during development is not well understood. Here, we use a *Drosophila* neuropil structure called the MB to address this issue. All three MB neuronal type refrain from crossing the midline. We found that glial-derived TGF-β signals bind to MB α/β neurons to mediate their midline stopping. Thus, we have identified the source of the patterning signal and its identity.

## Data Availability Statement

This manuscript contains previously unpublished data. Previously published stocks and their source are mentioned in the “Materials and Methods” section.

## Author Contributions

NM-K and IG designed, performed and analyzed experiments and wrote the manuscript. NI-Z provided technical assistance and performed specific experiments. OS designed and analyzed experiments, wrote the manuscript and procured funding.

## Conflict of Interest

The authors declare that the research was conducted in the absence of any commercial or financial relationships that could be construed as a potential conflict of interest.

## Supplementary Material

FIGURE S1 Midline crossing ranking, *plum* locus and CRISPR *plum*^V11X^ description, relates to Figure 1. **(A)** Examples of midline crossing severity ranking, for scores 1 **(A1)**, 3 **(A2)** and 5 **(A3)**. for more information, see “Materials and Methods” section.** (B)** Scheme of *plum* locus including available mutant and gRNAs used for generating *plum*^V11X^. **(C,D)** Confocal Z-projections of **(C)** WT or **(D)** *plum*^V11X^ MARCM neuroblast clones labeled with 201Y-Gal4. Yellow arrowheads indicate severe pruning defects. CD8::GFP (green); FasII (magenta). **(E,F)** Confocal Z-projections of WT **(E)** or *plum*^V11X^ **(F)** brains. Dashed red lines demarcate the extent of the β-lobes. FasII (gray); Scale bars, 20 μm. **(G)** Quantification of midline crossing phenotypes in **(E,F)**. *p*-value: ****p* < 0.001. Genotypes: **(C)** y, w, hsFlp/Y or +; CD8::GFP, 201Y/+; 82B, Gal80/82B (*n* = 6). **(D)** y, w, hsflp/Y or +; CD8::GFP, 201Y/+; 82B, Gal80/82B, plum^V11X^ (*n* = 21). **(E)** 201Y, CD8:GFP/+ (*n* = 45). **(F)** 82B, plum^V11X^ (*n* = 13). *p*-value: ****p* < 0.001.

FIGURE S2 *plum* RNAi in all MB neurons, relates to Figure 3. **(A–D)** Confocal Z-projections or selected slices, as depicted in the figure, of **(A,C)** brains expressing CD8::GFP or **(B,D)** those additionally expressing plum^RNAi^. Dashed red lines demarcate the extent of the β or β’-lobes. CD8::GFP (green); FasII (in **A’,B’**, gray); TRIO (in **C’,D’**, gray). Scale bars, 20 μm. **(E)** Quantification of the midline crossing phenotypes in **(A,B)**. *p*-values: ****p* < 0.001. Genotypes: **(A,C)** CD8/+; OK107/+ (*n* = 11, 20, respectively). **(B,D)** UAS- plum^RNAi^/CD8::GFP; OK107/+ (*n* = 19, 21, respectively).

FIGURE S3 Expression pattern of α’/β’ and α/β-specific Gal4s, relates to Figure 3. **(A–F)** Confocal Z-projections of WT brains expressing CD8::GFP driven by either c305a-Gal4 **(A–C)** or R44E04-Gal4 **(D–F)** driver at third-instar (L3) larva **(A,D)**, 0 h APF pupa **(B)**, 30 h APF pupa **(E)** and adult brains **(C,F)**. CD8::GFP (green); FasII (magenta). Scale bars, 20 μm. Genotypes: **(A–C)** c305a-Gal4/+; CD8::GFP/+. **(D–F)** CD8::GFP/+; R44-Gal4/+.

FIGURE S4 No midline crossing in controls expressing cell type specific Gal4 drivers, relates to Figure 3. **(A–C)** Confocal Z-projections of WT brains expressing CD8::GFP with the γ-specific R71G10 driver **(A)**, α/β-specific R44E04 driver **(B)**, or α’/β’-specific c305a driver **(C)**. Dashed red lines demarcate the extent of the β or β’-lobes. CD8::GFP (green in **A1–C1** and **A2–C2**), FasII (in **A1’–C1’**, gray), TRIO (in **A2’–C2’**, gray). Scale bars, 20 μm. Genotypes: **(A)** CD8::GFP/+; R71/+ (*n* = 16, 16, respectively). **(B)** c305a/+; CD8::GFP/+ (*n* = 28, 20, respectively). **(C)** CD8::GFP/+; R44/+ (*n* = 38, 20, respectively).

FIGURE S5 Plum over-expression within MB neurons does not affect β’-lobe length, relates to Figure 4. **(A,B)** Confocal Z-projections of **(A)** WT brains expressing CD8::GFP and **(B)** WT brains expressing CD8::GFP as well as Plum^FL^ driven by OK107-Gal4. Arrowheads in **(B1)** depict aberrant growth of β-lobes. CD8::GFP (green, **A1–B1, A3–B3**); TRIO (gray, **A2–B2**, magenta, **A3–B3**). Scale bars, 20 μm. Genotypes: **(A)** CD8::GFP/+;; OK107/+ (*n* = 20). **(B)** CD8::GFP/Plum^FL^;; OK107/+ (*n* = 5).

FIGURE S6 Plum’s intracellular, extracellular and transmembranal domains are required to induce midline stopping, relates to Figure 5. **(A–D)** Confocal Z-projections of adult *plum*^Δ1^ brains expressing CD8::GFP driven by the R44-Gal4 driver, and additionally expressing **(A)** UAS-Plum^Δcyt^, **(B)** UAS-Plum^ΔIG1-4^, **(C)** UAS-Plum^ΔIG1-3^, or **(D)** UAS-Plum^ΔECD-TM:CD8^. **(E)** Quantification of β-lobe midline crossing phenotypes in **(B–E)**. *p*-value: **p* = 0.017. ns = not significant. Dashed red lines demarcate the extent of the β-lobes. FasII (gray). Scale bars, 20 μm. Genotypes: **(A)** UAS-Plum^Δcyt^/+; 82B, plum^Δ1^/R44E04-Gal4, 82B, plum^Δ1^ (*n* = 18). **(B)** UAS-Plum^ΔIG1-4^/+; 82B, plum^Δ1^/R44E04-Gal4, 82B, plum^Δ1^ (*n* = 10). **(C)** UAS-Plum^ΔIG1-3^/+; 82B, plum^Δ1^/R44E04-Gal4, 82B, plum^Δ1^ (*n* = 17). **(D)** UAS-Plum^ΔECD-TM:CD8^/+; 82B, plum^Δ1^/R44E04-Gal4, 82B, plum^Δ1^ (*n* = 23). (Additionally, quantified in **E**): R44E04-Gal4, 82B, plum^Δ1^/82B, plum^Δ1^ (*n* = 39). UAS-Plum^FL^/+; 82B, plum^Δ1^/R44E04-Gal4, 82B, plum^Δ1^ (*n* = 20).

FIGURE S7 RhoGEF2 does not cause retraction when expressed in α/β neurons, relates to Figure 5. **(A)** Confocal Z-projection of adult brain expressing UAS-RhoGEF2 with the R44E04-Gal4 driver. Genotype: **(A)** R44E04-Gal4/UAS-RhoGEF2 (*n* = 17).
