# Supplementary figures and images for: Glial Derived TGF-β Instructs Axon Midline Stopping
Source: Front Mol Neurosci. 2019 Sep 27;12:232. doi: 10.3389/fnmol.2019.00232 (PMC6776989; doi:10.3389/fnmol.2019.00232)

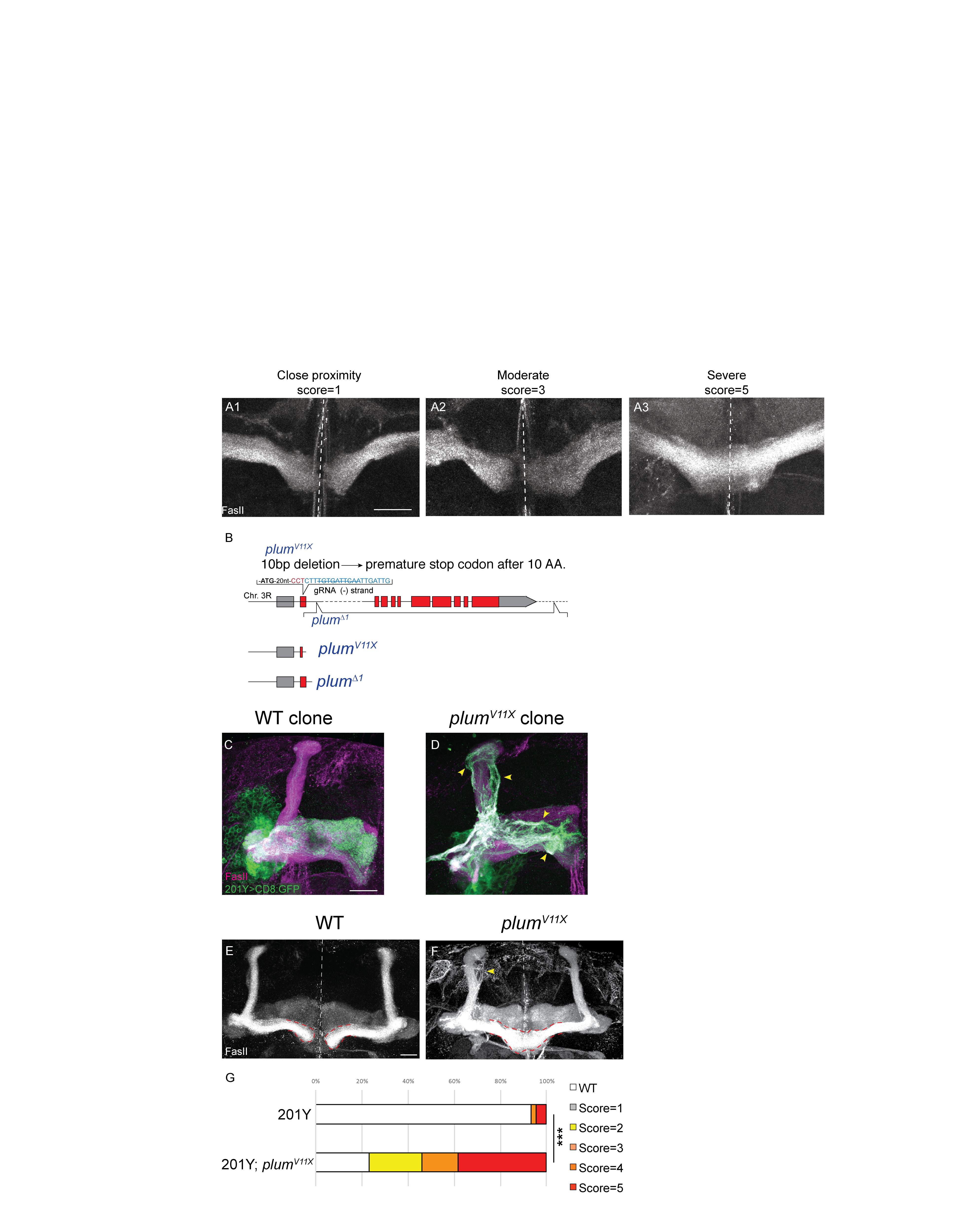

Supplement: FIGURE S1 — Midline crossing ranking, plum locus and CRISPR plumV11X description, relates to Figure 1. (A) Examples of midline crossing severity ranking, for scores 1 (A1), 3 (A2) and 5 (A3). for more information, see “Materials and Methods” section. (B) Scheme of plum locus including available mutant and gRNAs used for generating plumV11X. (C,D) Confocal Z-projections of (C) WT or (D) plumV11X MARCM neuroblast clones labeled with 201Y-Gal4. Yellow arrowheads indicate severe pruning defects. CD8::GFP (green); FasII (magenta). (E,F) Confocal Z-projections of WT (E) or plumV11X (F) brains. Dashed red lines demarcate the extent of the β-lobes. FasII (gray); Scale bars, 20 μm. (G) Quantification of midline crossing phenotypes in (E,F). p-value: ***p < 0.001. Genotypes: (C) y, w, hsFlp/Y or +; CD8::GFP, 201Y/+; 82B, Gal80/82B (n = 6). (D) y, w, hsflp/Y or +; CD8::GFP, 201Y/+; 82B, Gal80/82B, plumV11X (n = 21). (E) 201Y, CD8:GFP/+ (n = 45). (F) 82B, plumV11X (n = 13). p-value: ***p < 0.001. [file Image_1.TIF]

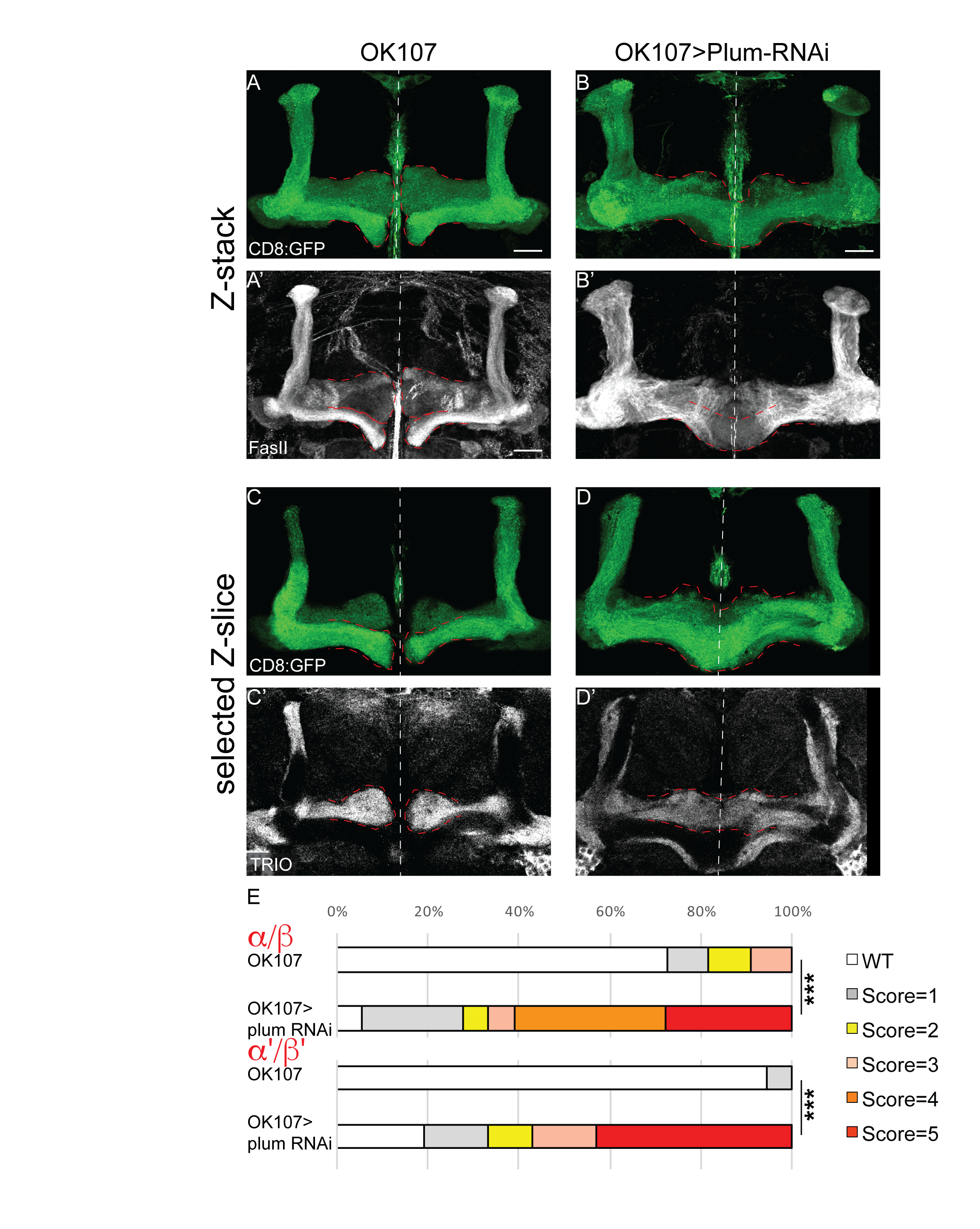

Supplement: FIGURE S2 — plum RNAi in all MB neurons, relates to Figure 3. (A–D) Confocal Z-projections or selected slices, as depicted in the figure, of (A,C) brains expressing CD8::GFP or (B,D) those additionally expressing plumRNAi. Dashed red lines demarcate the extent of the β or β’-lobes. CD8::GFP (green); FasII (in A’,B’, gray); TRIO (in C’,D’, gray). Scale bars, 20 μm. (E) Quantification of the midline crossing phenotypes in (A,B). p-values: ***p < 0.001. Genotypes: (A,C) CD8/+; OK107/+ (n = 11, 20, respectively). (B,D) UAS- plumRNAi/CD8::GFP; OK107/+ (n = 19, 21, respectively). [file Image_2.TIF]

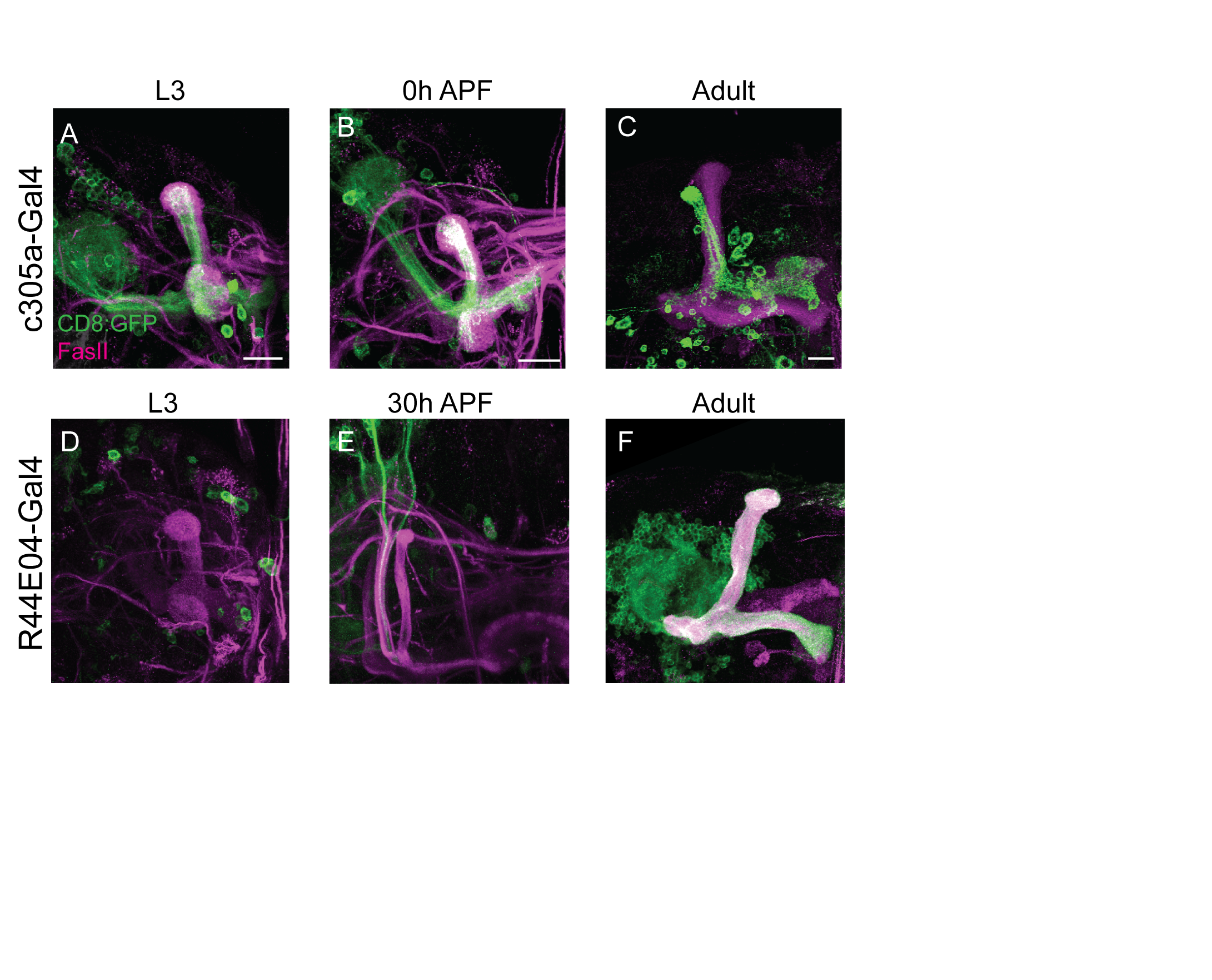

Supplement: FIGURE S3 — Expression pattern of α’/β’ and α/β-specific Gal4s, relates to Figure 3. (A–F) Confocal Z-projections of WT brains expressing CD8::GFP driven by either c305a-Gal4 (A–C) or R44E04-Gal4 (D–F) driver at third-instar (L3) larva (A,D), 0 h APF pupa (B), 30 h APF pupa (E) and adult brains (C,F). CD8::GFP (green); FasII (magenta). Scale bars, 20 μm. Genotypes: (A–C) c305a-Gal4/+; CD8::GFP/+. (D–F) CD8::GFP/+; R44-Gal4/+. [file Image_3.TIF]

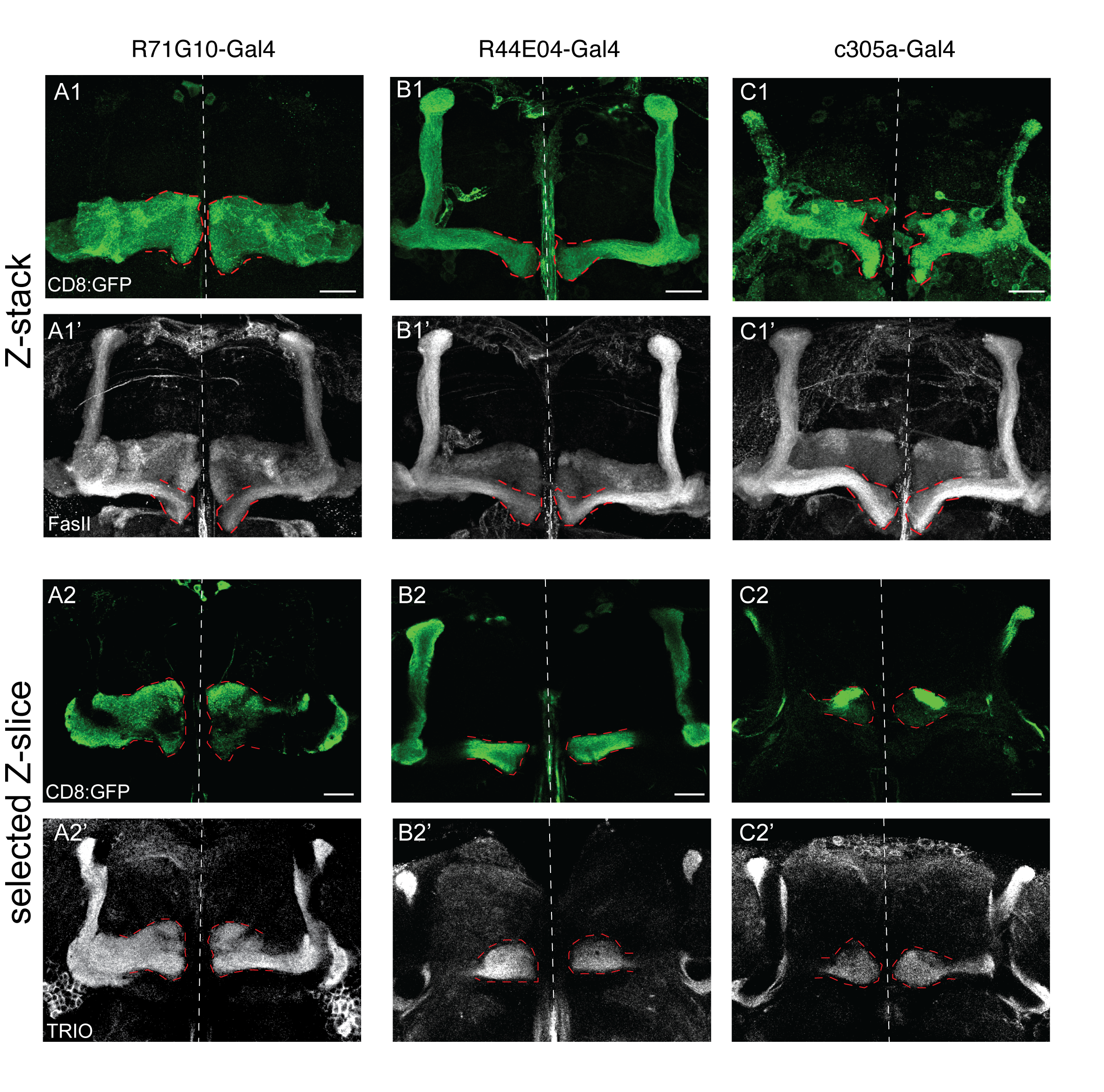

Supplement: FIGURE S4 — No midline crossing in controls expressing cell type specific Gal4 drivers, relates to Figure 3. (A–C) Confocal Z-projections of WT brains expressing CD8::GFP with the γ-specific R71G10 driver (A), α/β-specific R44E04 driver (B), or α’/β’-specific c305a driver (C). Dashed red lines demarcate the extent of the β or β’-lobes. CD8::GFP (green in A1–C1 and A2–C2), FasII (in A1’–C1’, gray), TRIO (in A2’–C2’, gray). Scale bars, 20 μm. Genotypes: (A) CD8::GFP/+; R71/+ (n = 16, 16, respectively). (B) c305a/+; CD8::GFP/+ (n = 28, 20, respectively). (C) CD8::GFP/+; R44/+ (n = 38, 20, respectively). [file Image_4.TIF]

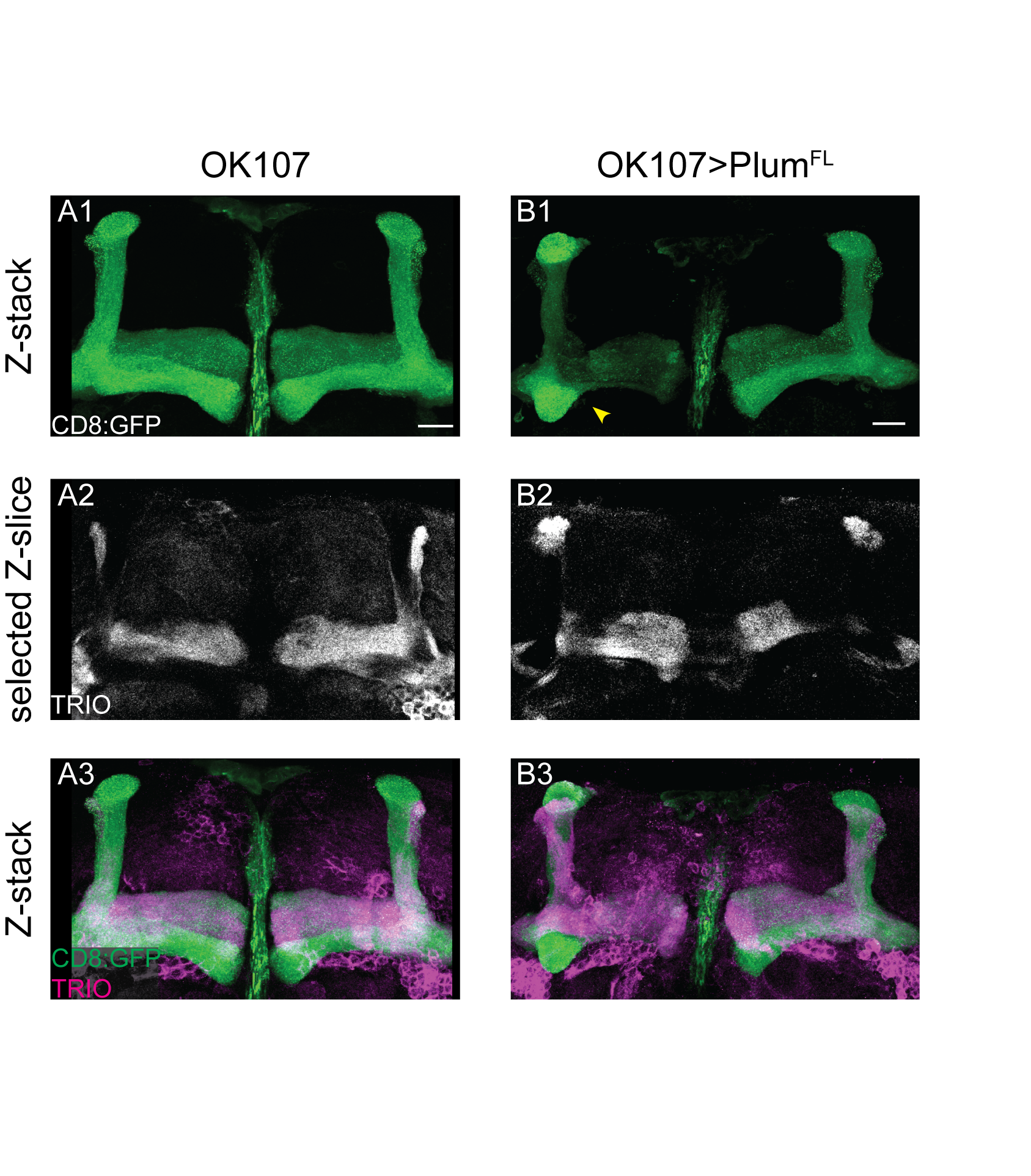

Supplement: FIGURE S5 — Plum over-expression within MB neurons does not affect β’-lobe length, relates to Figure 4. (A,B) Confocal Z-projections of (A) WT brains expressing CD8::GFP and (B) WT brains expressing CD8::GFP as well as PlumFL driven by OK107-Gal4. Arrowheads in (B1) depict aberrant growth of β-lobes. CD8::GFP (green, A1–B1, A3–B3); TRIO (gray, A2–B2, magenta, A3–B3). Scale bars, 20 μm. Genotypes: (A) CD8::GFP/+;; OK107/+ (n = 20). (B) CD8::GFP/PlumFL;; OK107/+ (n = 5). [file Image_5.TIF]

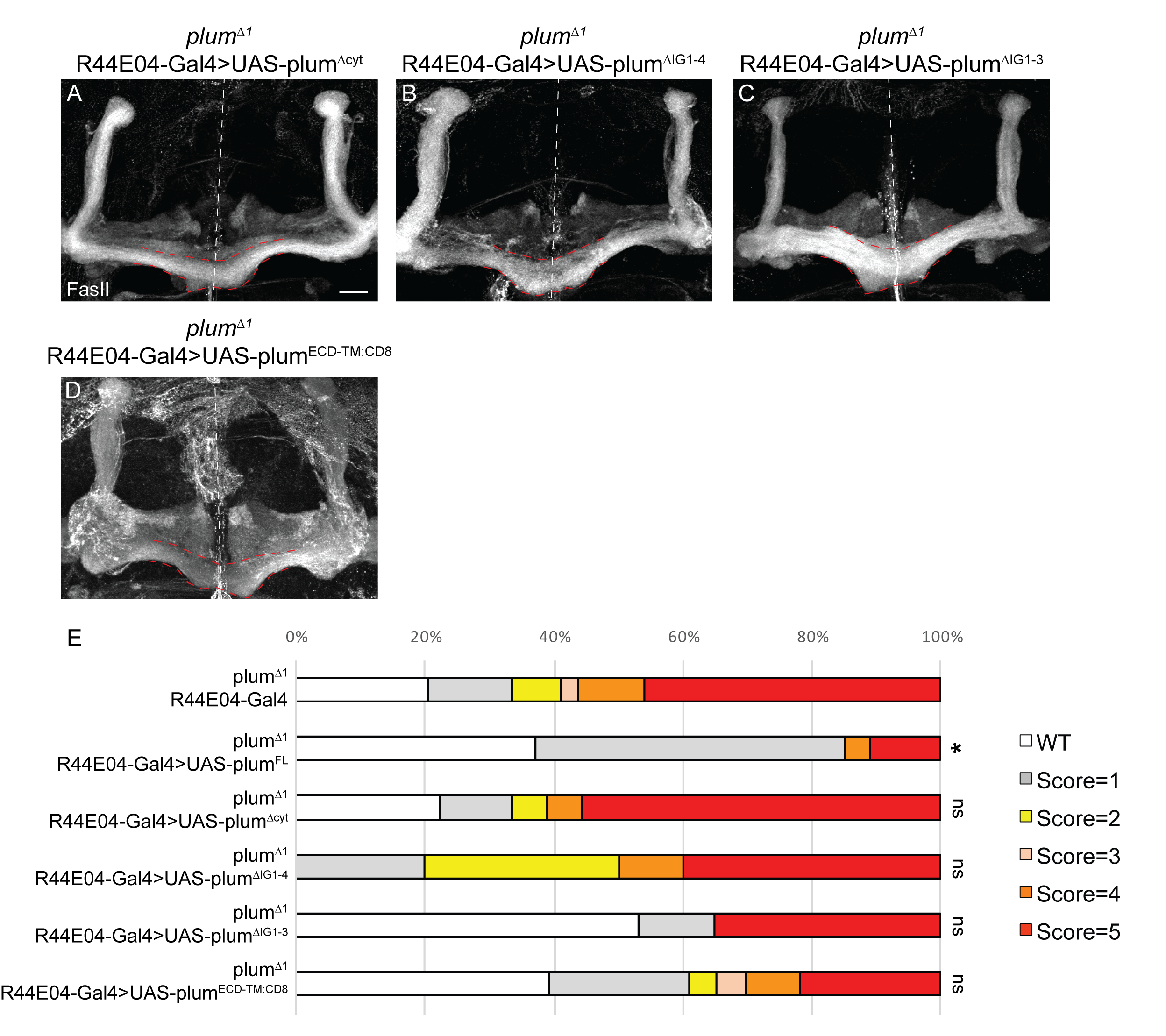

Supplement: FIGURE S6 — Plum’s intracellular, extracellular and transmembranal domains are required to induce midline stopping, relates to Figure 5. (A–D) Confocal Z-projections of adult plumΔ1 brains expressing CD8::GFP driven by the R44-Gal4 driver, and additionally expressing (A) UAS-PlumΔcyt, (B) UAS-PlumΔIG1-4, (C) UAS-PlumΔIG1-3, or (D) UAS-PlumΔECD-TM:CD8. (E) Quantification of β-lobe midline crossing phenotypes in (B–E). p-value: *p = 0.017. ns = not significant. Dashed red lines demarcate the extent of the β-lobes. FasII (gray). Scale bars, 20 μm. Genotypes: (A) UAS-PlumΔcyt/+; 82B, plumΔ1/R44E04-Gal4, 82B, plumΔ1 (n = 18). (B) UAS-PlumΔIG1-4/+; 82B, plumΔ1/R44E04-Gal4, 82B, plumΔ1 (n = 10). (C) UAS-PlumΔIG1-3/+; 82B, plumΔ1/R44E04-Gal4, 82B, plumΔ1 (n = 17). (D) UAS-PlumΔECD-TM:CD8/+; 82B, plumΔ1/R44E04-Gal4, 82B, plumΔ1 (n = 23). (Additionally, quantified in E): R44E04-Gal4, 82B, plumΔ1/82B, plumΔ1 (n = 39). UAS-PlumFL/+; 82B, plumΔ1/R44E04-Gal4, 82B, plumΔ1 (n = 20). [file Image_6.TIF]

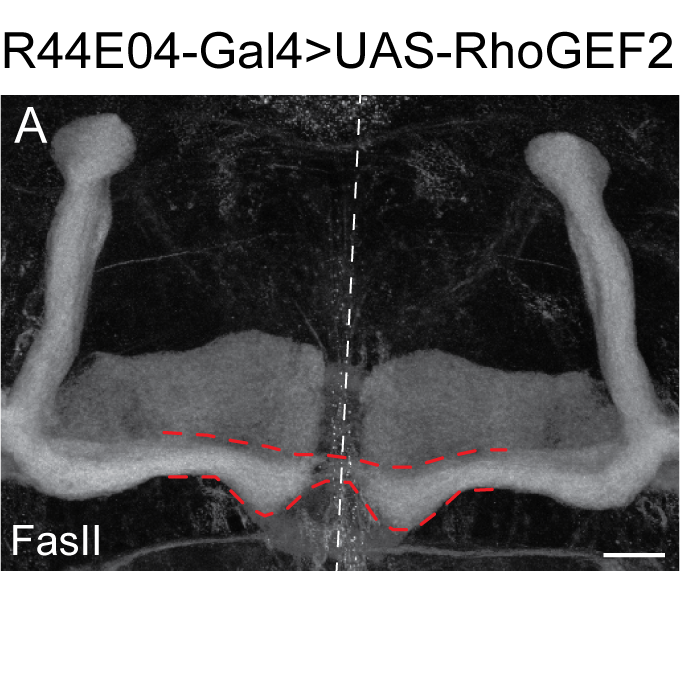

Supplement: FIGURE S7 — RhoGEF2 does not cause retraction when expressed in α/β neurons, relates to Figure 5. (A) Confocal Z-projection of adult brain expressing UAS-RhoGEF2 with the R44E04-Gal4 driver. Genotype: (A) R44E04-Gal4/UAS-RhoGEF2 (n = 17). [file Image_7.TIF]
